# Application of FRAME for Simultaneous LIF and LII Imaging in Sooting Flames Using a Single Camera

**DOI:** 10.3390/s20195534

**Published:** 2020-09-27

**Authors:** Yogeshwar Nath Mishra, Prasad Boggavarapu, Devashish Chorey, Lars Zigan, Stefan Will, Devendra Deshmukh, Ravikrishna Rayavarapu

**Affiliations:** 1 Institute of Engineering Thermodynamics, Friedrich-Alexander University (FAU), 91058 Erlangen, Germany; lars.zigan@fau.de (L.Z.); stefan.will@fau.de (S.W.); 2 Discipline of Mechanical Engineering, Indian Institute of Technology, Indore 453552, India; phd1801203001@iiti.ac.in (D.C.); dldeshmukh@iiti.ac.in (D.D.); 3 NASA-Jet Propulsion Laboratory, California Institute of Technology, Pasadena, CA 91109, USA; 4 Department of Mechanical Engineering, Indian Institute of Science, Bangalore 560012, India; bvvsv.prasad@gmail.com (P.B.); ravikris@iisc.ac.in (R.R.); 5 Erlangen Graduate School in Advanced Optical Technologies, FAU, 91054 Erlangen, Germany

**Keywords:** structured illumination, multispectral imaging, flame species, laser-Induced fluorescence, laser-induced incandescence, soot, combustion

## Abstract

In this article, the application of the FRAME (Frequency Recognition Algorithm for Multiple Exposures) technique is presented for multi-species measurements in symmetric and asymmetric ethylene/air diffusion flames. Laminar Bunsen-type and swirled diffusion flames are investigated to gain a better understanding of sooting combustion. For this purpose, simultaneous imaging is conducted in terms of Laser-Induced Fluorescence (LIF) of Polycyclic Aromatic Hydrocarbons (PAH) and Laser-Induced Incandescence (LII) of soot particles. Subsequently, the approach is utilized for simultaneous imaging of hydroxyl (OH)-LIF and soot-LII. Here, the modulated LIF- and LII-signals are acquired together as a single sub-image—with a single exposure utilizing the full sensor size of a single camera. By employing the frequency-recognition algorithm on the single image, the LIF- and LII-signals are spectrally isolated—generating two individual LIF- and LII-images. The flame luminosity and out-of-focus light such as reflected surrounding laser light are detected as non-modulated signals in the unprocessed image. These unwanted signals are suppressed using the image post-processing, and, therefore, the image contrast of the two resulting images is improved. It is found that PAHs mainly exist in the inner region near the burner and are surrounded by soot. The majority of the OH is distributed on the outer edges of the flame—representing the reaction zone and soot-oxidation region of the flame.

## 1. Introduction

During the combustion of hydrocarbon fuels, multiple intermediate products are formed such as hydroxyl (OH), formaldehyde (CH_2_O), Polycyclic Aromatic Hydrocarbons (PAHs), CH, CO, etc. [1], which depend on the stoichiometry of the flame. Intermediate products such as OH, CH_2_O, CH, and CO control the heat release rate of a flame. Furthermore, soot is produced under fuel-rich conditions, depending on the concentration of PAHs. Therefore, the “footprint” of these chemical species can be used to track the combustion and post-combustion processes [2,3]. For instance, OH is one of the most important intermediates in flames of hydrocarbon fuels for validation of reaction mechanism and chemical kinetic models [4,5,6]. The species CH_2_O and OH can be utilized for visualization of flame fronts and the heat release zone [7,8]. Species such as PAHs and soot particles are harmful pollutants generated from the combustion of fuels [9,10,11,12,13,14,15]. Due to the stricter emission norms, much attention has been given to the further understanding of the combustion chemistry, and especially the soot formation. Nevertheless, quantitative measurements in flames are challenging, as simultaneous multi-species measurements are required [3,14]. A variety of optical measurement techniques have been reported to measure single and multi-species in flames [2], e.g., light scattering, absorption, and fluorescence spectroscopy. For planar measurements, Laser Sheet Imaging (LSI) is one of the most established diagnostics in combustion [16]. In LSI, a thin sheet of laser is formed, which intersects a plane in the flame to image a two-dimensional map usually on a centimeter’s scale. Moreover, species in the flame can be preferably resolved both spatially and temporally [16,17]. Planar Laser-Induced Fluorescence (PLIF) is commonly employed for two-dimensional mapping of the relative concentration of species in flames. Since the 1980s, PLIF has been used as a highly sensitive technique to preferably visualize the combustion fields, capturing rapid changes of the flame structures and combustion process, temperature mapping, and qualitative as well as quantitative (mainly at low-pressure conditions) imaging of radical concentrations [18,19]. When a radical or molecule in the flame is excited by a laser (usually pulsed) of a specific wavelength, its low energy state (usually ground state) is changed to a higher energy state (excited state). While relaxing back from excited to the ground state, the energy difference between the two states may be released in form of photons (fluorescence). The Planar Laser-Induced Incandescence (PLII) imaging is another common LSI method to measure the soot volume fraction of mature-soot particles or black carbon in combustion [11,15,20]. In PLII, the soot particles are heated to sufficiently high temperatures with high-fluence pulsed lasers to evoke increased black-body radiation. With a spatially-resolved detection of the emitted signal, soot volume fraction mapping is performed with additional time-resolved detection, i.e., capturing images at various delays after the laser pulse primary particle sizing becomes feasible [15].

For simultaneously imaging multiple species in flames, a combination of multiple lasers and several cameras (which are time-gated and/or equipped with different spectral filters) is required—making the optical setup complex and resource-intensive [21,22,23,24]. For example, Sjöholm et al. [21] utilized three pulsed lasers to generate four different wavelengths of 266 nm, 309 nm, 355 nm, and 431 nm to excite fluorescence of toluene, OH, CH_2_O, and CH, respectively, in a methane jet flame. For simultaneous PLIF of those four different species, four intensified CCD camera systems with respective objectives were utilized. Moreover, several beam splitters and optical filters were applied to separate the signals and minimize the spectral overlap. In another study, Zhou et al. [22] combined four lasers at 253 nm for HCO excitation, 283 nm for OH-PLIF, 355 nm for probing CH_2_O, and 387 nm for CH excitation, respectively, in turbulent premixed methane/air flames. Three intensified CCD cameras were used for simultaneous multi-species measurements. Franzelli et al. [23] performed simultaneous imaging of soot, PAH, and OH in a turbulent methane diffusion flame using 284 nm as the excitation wavelength of a dye laser and three high-speed intensified CMOS cameras. In a recent study, Mulla et al. [24] conducted instantaneous soot-PLII and PLIF of PAH and OH in a turbulent n-heptane jet spray flame. The optical setup consisted of two pulsed lasers at 283 nm (to excite both PAH- and OH-LIF) and 1064 nm (for LII) and three intensified CCD cameras including two pulse generators to synchronize the lasers and cameras. Two light sheets were formed and overlaid in opposite directions of their propagations. The OH-PLIF and soot-LII cameras were facing each other opposite on either side of the laser sheet, while the PAH-LIF camera was kept off-normal using a Scheimpflug adaptor. Thus, LSI setups for simultaneous multispecies detection are not easy to establish and demand proper alignment and expertise apart from many resource-intensive lasers sources and detectors.

Furthermore, despite mapping multiple species in a plane, all the “conventional” LSI measurements described above are often perturbed by issues such as stray light and other background radiation. Stray light is an undesired interference signal comprising of multiple reflections, scattering, laser-induced backgrounds, flame luminosity, black body radiation [25], and spectral interference of multi optical signals (i.e., fluorescence) [14]. In LSI, the generation of stray light is pronounced mainly due to the larger area of the sample illuminated by the laser sheet and also the large detection angle of the detector kept at 90° to the incident plane [26,27]. Therefore, stray light interferences contribute to erroneous quantitative imaging [27], especially in challenging combustion environments such as in optically-accessible IC engines, gas turbines, or burners. To generate high fidelity LSI measurement data, a precise subtraction of stray light (background) from the signal of interest is required. To that end, for more than a decade, SLIPI (Structured Laser Illumination Planar Imaging) has been used to suppress background signals and multiple light scattering in non-combusting optically dense sprays [28,29,30], and in flames with distinct background and stray light [26,27]. For example, using SLIPI, optically dense diesel sprays [31] and ethanol sprays were characterized in terms of spray structures and spray quantities [29]. The averaged SLIPI [26] and single-shot SLIPI techniques [27] were employed in combination with Rayleigh thermometry in a high background scattering environment for full-field temperature measurement in methane/air flames. It is demonstrated in an earlier work [27] that due to strong background interference, unsatisfactory results with “conventional”-Rayleigh thermometry in flames were found, whereas SLIPI-Rayleigh thermometry still maintains both the symmetry and the value of the absolute temperature. In contrast to “conventional” LSI, the light sheet in SLIPI is superimposed with a spatial modulation—usually sinusoidal [28]. This sinusoidal modulation acts as a “fingerprint” for identifying the singly scattered photons within the plane of the laser sheet illuminating the sample. Therefore, it becomes possible to distinguish between the singly scattered and multiply scattered photons by the post-processing of the modulated image [28]. To preserve the full spatial resolution of the image, the standard averaged SLIPI technique (Three-phase SLIPI: 3p-SLIPI) requires the recording of at least three spatially-modulated images (referred to as sub-images) [28]. However, a single-shot SLIPI image reconstruction using only one sub-image (One-phase SLIPI: 1p-SLIPI) is also possible albeit at the cost of a (minor) loss in image spatial resolution in spray images as demonstrated in a previous work [32]. A nearly unnoticeable loss in spatial resolution can be achieved in flames, provided that the frequency of incident modulation is generally more than two times higher in 1p-SLIPI [27] in comparison to 3p-SLIPI [26].

**Figure 1:**
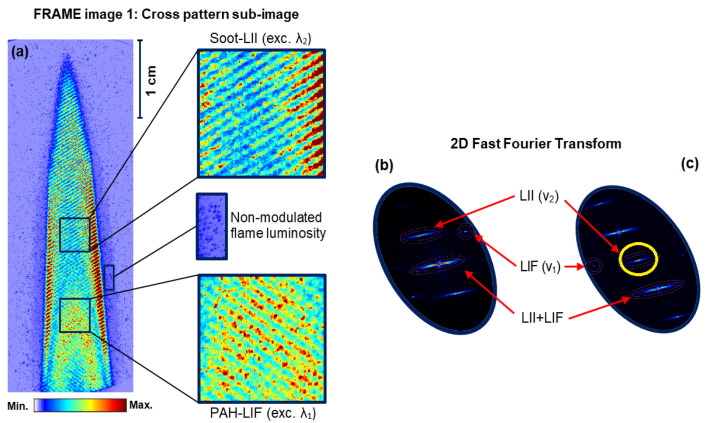
(**a**) FRAME image 1: single-shot unprocessed sub-image of both PAH-LIF and soot-LII signals recorded using a single camera. Zoomed view: The LIF and LII signals generated by two laser excitations (λ_1_, λ_2_) are modulated and flame luminosity including stray lights are non-modulated signals. (**b**) Two-dimensional (2D) FFT of the image in (**a**), showing two signals appearing at different locations in the frequency domain (Fourier space). In (**c**), 2D FFT of a resulting image generated from the multiplication of sub-image data in (**a**) and a sinusoidal reference signal of spatial frequency (ν_2_).

Keeping in mind the issues of stray light and the cost and complexity of the optical setups mentioned above, recently, the SLIPI-based approach known as FRAME (Frequency Recognition Algorithm for Multiple Exposures) was demonstrated for multispecies two-dimensional (2D) imaging in flames [33]. In FRAME, the sample is excited by multiple lasers and the corresponding optical signals are recorded on a single camera—using a single acquisition and utilizing the full sensor area. The use of a single camera and single objective ensures the same field-of-view and light collection angle, which in the optical setups mentioned above requires high efforts in alignment and later pixel-by-pixel overlapping of field-of-views of more than one camera. However, in FRAME, it comes at the cost of (usually a very minute) loss in spatial resolution as mentioned above. Developed by Kristensson et al., the FRAME technique combines 1p-SLIPI (of high modulation frequency—from 10 to 30 line-pairs/mm Ronchi gratings) and multispectral imaging, where the LIF signals of two or more probed species are recorded simultaneously [33,34]. For instance, in non-combusting media, four modulated continuous lasers of wavelengths 405 nm, 447 nm, 450 nm, and 532 nm were combined to perform multispectral imaging of the dynamic samples of fluorophores on a single camera [34]. Li et al. [33] employed the method on a turbulent dimethyl ether/air flame using two modulated pulsed laser sheets—one at 283 nm was exciting OH-LIF, while another at 355 nm was exciting CH_2_O-LIF, simultaneously. Therefore, unlike single laser sheets used in 1p-SLIPI, in FRAME, two or more laser sheets are utilized for probing the sample. Moreover, these multiple laser sheets are reasonably aligned (see Figure 1) or rearranged in the sample illumination plane so that they can be recognized later during the FRAME image post-processing using a frequency-sensitive algorithm [33].

In this investigation, we present a modified setup of FRAME to simplify the optical design in [33], which improves the setup’s practicality in terms of minimizing the loss of the laser fluence. The combination of a square Ronchi grating and a spatial filter used for generating a sinusoidal modulation in [33] is replaced here by a custom-made transmission grating of quartz (fabrication procedure is mentioned elsewhere [30]). Using the modified FRAME setup, we demonstrate, for the first time: (i) simultaneous PAH-LIF and soot-LII imaging and (ii) simultaneous OH-LIF and soot-LII imaging using a single camera, sequentially. Here, first, an axisymmetric ethylene/air laminar diffusion flame generated by a Gülder burner [35,36,37,38] is investigated. Then, an asymmetric flame is generated by fixing a swirler on top of the burner nozzle. In the literature, the laminar diffusion flames of hydrocarbon fuels have been extensively investigated to gain a better understanding of soot production and oxidation mechanisms. Here, however, only the qualitative distribution of each probed species is presented to demonstrate the application of the FRAME technique as the first step towards a detailed analysis.

## 2. Frequency Recognition Algorithm for Multiple Exposures (FRAME)

**Figure 2:**
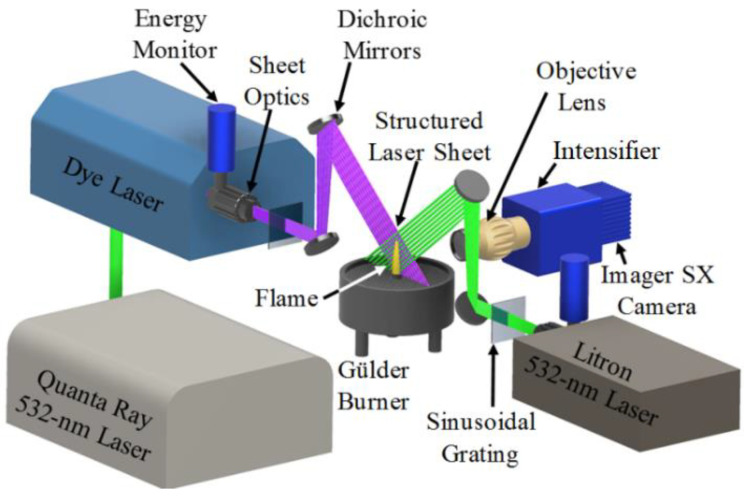
Schematic of the FRAME setup: two pulsed laser sources are converted into laser sheets and aligned in cross-illumination configuration using dichroic mirrors. Two custom made sinusoidal gratings of 10 line-pairs/mm create the spatial modulation. An optical filter, UV objective, intensifier and sCMOS camera are used for simultaneous LIF and LII imaging in ethylene/air diffusion flames of a Gülder burner.

Figure 1a shows an unprocessed single-shot FRAME image of the modulated signals of interest—PAH-LIF and soot-LII signals, and non-modulated background signals, i.e., stray light and flame luminosity, along with their zoomed view. The image is recorded using the experimental setup given in Figure 2. Note that the flame luminosity signal is retained here, which in the conventional detection is usually subtracted as a background from the signal of interest. This non-modulated flame luminosity light is suppressed naturally during the image post-processing, which only extracts the amplitude of the modulation—signal of interest. The cross-patterned sub-image in Figure 1a consists of two modulated wavelength components of 283.5 nm wavelength (λ_1_) that excites the fluorescence of PAHs (and OH, see below, but this radical is not detected with the filter used) and another laser at 532 nm (λ_2_) excites incandescence in the soot particles in flame.

Under conventional LSI (without modulation and coplanar illumination), both signals would appear merged (excited by λ_1_ + λ_2_) on the same detector and cannot be distinguished. In contrast, in the FRAME image, both “coded” laser sheets are aligned orthogonal to each other in Figure 1a. With this orthogonal geometry, the corresponding signals appear at different locations in the 2D Fourier transform or frequency domain—as ν_1_ and ν_2_ in Figure 1b. Therefore, both signals can be acquired on a single image utilizing the full camera pixels, and the recording of two separate images is not needed. The two signals are distinguished from each other in data processing by a signal-processing algorithm known as frequency-sensitive spatial lock-in detection [27,33,39,40,41]. According to the algorithm, the intensity of image Figure 1a can be expressed in one dimension (1D) as:

(1) $$I\;=S_{1}(1+\sin\left(2{\pi \nu }_{1}+\varphi_{1}\right))+S_{2}\;\left(1+\sin\left(2{\pi \nu }_{2}+{\;\varphi }_{2}\right)\right)$$

where I (x) or I (y) is the acquired cross-patterned image, $S_{1}$ and $S_{2}\;$ are the two sample responses, and ν  and φ are the spatial frequency and spatial phase, respectively. From the 2D FFT of the image in Figure 1b, it is seen that in the reciprocal space both modulations of PAH-LIF (ν_1_) and soot-LII (ν_2_) appear as isolated peaks (also referred to as 1st order peaks), while the non-modulated image components along with (LIF + LII) appear at the center of the Fourier space, as the 0th order peak. In Figure 1c, the resulting FFT of an image of intensity (I_1_) generated from matrix multiplication between raw data in Figure 1a and a reference signal (R_1_)—a sine wave of spatial frequency ν_2_—is given. Similarly, a second image of intensity (I_2_) (not shown here) is also generated by multiplying the image in Figure 1a with a reference signal (R_2_), which is phase-shifted by 90° to R_1_. When comparing (b) and (c), it can be seen in (c) that the 1st order and 0th order switch places. Now, to extract the soot-LII signal (ν_2_ components), a low pass filter (filter size σ = 0.12, rotational symmetric Gaussian filter: indicated as a yellow circle in (c)) is applied on two resulting FFT images. The value of the filter sizes represents a percentage and the entire image area in the reciprocal space corresponds to the σ-value of 100% [33]. After the filtering process, the resulting intensities can be expressed in one dimension (1D) as:

(2) $${\tilde{I}}_{1}\left(x\right)=\tilde{I_{S}}\sin\left(\phi \right)\;$$

(3) $${\tilde{I}}_{2}\left(x\right)=\tilde{I_{S}}\cos\left(\phi \right)\;$$

where the tilde indicates the applied low-pass filters. From Equations (2) and (3), the resulting signal intensity can be calculated as:

(4) $$\tilde{I_{S}}=2\sqrt{{\left({\tilde{I}}_{1}\right)}^{2}+{\left({\tilde{I}}_{2}\right)}^{2}}\;\;\;\;\;\;\;\;\;\;\;\;\;\;\;$$

where the term $\tilde{I_{S}}$ corresponds to the amplitude of the modulation of the desired LII-signal (ν_2_ components), obtained after operating a low-pass Fourier filtering process. Similarly, a post-processing routine is employed to extract the desired LIF-signal (ν_1_ components). Finally, a conventional image carrying both LII + LIF signals can be extracted by applying the low-pass Fourier filtering process on the 0th order component of the FFT in Figure 1b.

## 3. Description of the Experiment

### 3.1. FRAME Optical Setup

The optical arrangement of the FRAME setup is given in Figure 2. The laser source (left side) is a dye laser (PrecisionScan, Sirah Lasertechnik GmbH, Grevenbroich, Germany, λ_1_: 283.5 nm, pulse width: 10 ns, rep. rate: 10 Hz), which is pumped by an Nd:YAG laser (Quanta Ray, Spectra-Physics Inc., Santa Clara, CA, USA, 532 nm, 10 Hz). The second laser on the right side (Nano-PIV, Litron lasers, Rugby, UK) is operated at λ_2_ = 532 nm (pulse width: 10 ns, rep. rate: 10 Hz). The two laser beams are converted to laser sheets using two similar sheet forming optics setups (Models: UV: VZ14-1033 and VIS: VZ10-0402, LaVision GmbH, Goettingen, Germany). Employing two dichroic mirrors on each side of the setup, it is possible to adjust and achieve a cross-patterned geometry between the two light sheets. As mentioned above, if both modulations arrive from the same plane and same direction, their corresponding spatial frequency components will overlap in the Fourier domain. Therefore, this orthogonal geometry helps in positioning the modulated components of two signals isolated from each in the Fourier domain (Figure 1b). It further assists in filtering the two signals during lock-in detection. Therefore, adequate isolation of two signals also depends on the alignment of the two-laser sheets and should be kept in mind while designing the FRAME setup. A detailed analysis of the effects of alignments of laser sheets on signal isolation in FRAME is given in [41]. Here, to monitor pulse-to-pulse variations of laser fluence, two identical energy meter setups (Models: VZ15-0070, LaVision GmbH, Goettingen, Germany) are kept just in front of the sheet optics setups. A top hat beam profile is achieved for each laser beam path by fixing an aperture in between the energy monitor setup and the sheet forming optics setup. For creating spatially modulated laser sheets, two custom-made transmission gratings—10 line-pairs/mm rulings, each fabricated on 2-inch quartz—are used. Details of the custom-made grating (withstanding high energy density of pulsed lasers) fabrication are provided elsewhere [30]. The gratings are directly introduced in the path of the laser sheet to create the spatial modulation unlike in a previous work [33], using a square Ronchi grating and a spatial filter in the path of the laser beam. This reduces the overall loss of initial laser energy to approximately 50% as compared to 85% in the original FRAME setup in [33]. Each laser beam is guided through two mirrors (each 2-inch diameter) to achieve a cross-patterned overlapped illumination between the two laser sheets (see Figure 1a). The overlapped region has an area of 50 × 50 mm^2^ and a laser sheet thickness of approximately 0.5 mm.

The averaged laser fluence at the incident plane for the 283.5 nm wavelength for OH excitation is approximately 12 mJ/cm^2^ The averaged laser fluence at the incident plane for the 532 nm laser for the soot excitation is ~275 mJ/cm^2^, which is within the plateau region [42], and in this range, the detected signal remains independent of small variations in the laser sheet profile. Laser extinction across the flame (which is seen here) usually does not result in the loss of the LII signal [43]. The pulse-to-pulse variation in laser energy for the 283.5 nm laser is around 7.5% and is within 1% for the 532 nm wavelength laser. Both laser pulses arrive at the same time (without delay), while the gating time of the intensifier is set to 350 ns (150 ns after the start of the laser pulse) throughout the experiments. The gating time is chosen to detect both fluorescence (which lasts a few ns) and incandescence signals (which lasts a few hundred ns).

The fluorescence and incandescence signals from the cross-patterned region are detected using a UV macroscopic objective (LaVision GmbH, Germany, f = 100 mm, f_#_ = 2.8), an image intensifier (5 ns gate, LaVision GmbH, Goettingen, Germany), and an sCMOS camera (Imager SX, 12 bit, 1776 × 2360 pixels, LaVision GmbH, Goettingen, Germany). Two lasers and the camera are synchronized using a programmable timing unit (PTUX, LaVision GmbH, Goettingen, Germany), and images are recorded using DaVis 8.1 software (LaVision GmbH, Goettingen, Germany). The total field-of-view captured by the camera image is 59 mm tall and 44.15 mm wide with a corresponding magnification of 40 pixels/mm. Using this setup, an axisymmetric and an asymmetric flame are probed (details in Section 3.1) and two sequential measurements are performed. The two laser sheets are pointed towards the edges of the burner nozzle and any reflections from the region are avoided by adjusting the alignment with the dichroic mirrors. Furthermore, any direct laser light reflections emerging from the air co-flow area is blocked by its outer edges towards the camera. In the first measurement, simultaneous PAH-LIF and soot-LII imaging is demonstrated on the central plane of the flame. Here, PAH fluorescence is excited by 283.5 nm (laser 1), while soot-LII is probed using 532 nm excitation (laser 2). Both LIF and LII signals are spectrally collected through a bandpass filter of bandwidth 360–430 nm (LaVision GmbH, Goettingen, Germany) fixed in front of the camera objective. The detection of PAH-LIF signal in the spectral range of 350–400 nm indicates the presence of smaller PAH (primarily of two to four-ringed) [23,24,44]. It is worth highlighting that the interpretation of PAH from their LIF signal and their isolation is not straightforward due to overlap between the excitation and emission spectra of different classes (rings) of PAH [11]. In the second measurement, simultaneous OH-LIF and soot-LII imaging is demonstrated. Here, the two laser wavelengths are kept unchanged, only the optical filter is changed, which is now a band-pass optical filter of bandwidth 305–315 nm (LaVision GmbH, Goettingen, Germany). The laser energy for OH-LIF is increased by ~11% compared to PAH-LIF excitation, while for LII the laser energy remains the same. However, the intensifier gain is reduced by nearly 5% here compared to the first measurement to avoid camera saturation. To detect OH fluorescence, the selected optical filter is well adapted and it is sufficiently narrow to reduce the interference with the PAH fluorescence signal, which occurs over a broader band of wavelengths (that is also excitable at 283.5 nm) [24]. The overall contribution of PAH-LIF to the optical signal in the spectral range of the optical filter is expected to be negligible compared to that of the OH-LIF signal. Therefore, PAH+soot, OH+soot measurements reported in this study are performed one after another and not simultaneously. Nevertheless, simultaneous PAH+OH+soot imaging can also be performed with the current setup either by (i) adding a second UV objective and a camera in combination with two optical filters, or (ii) by mounting an image doubler in front of the objective of the current setup—to split the camera sensor in two images, which may result in further reduction of pixel resolution.

### 3.2. Details of the Burner and Flames

**Figure 3:**
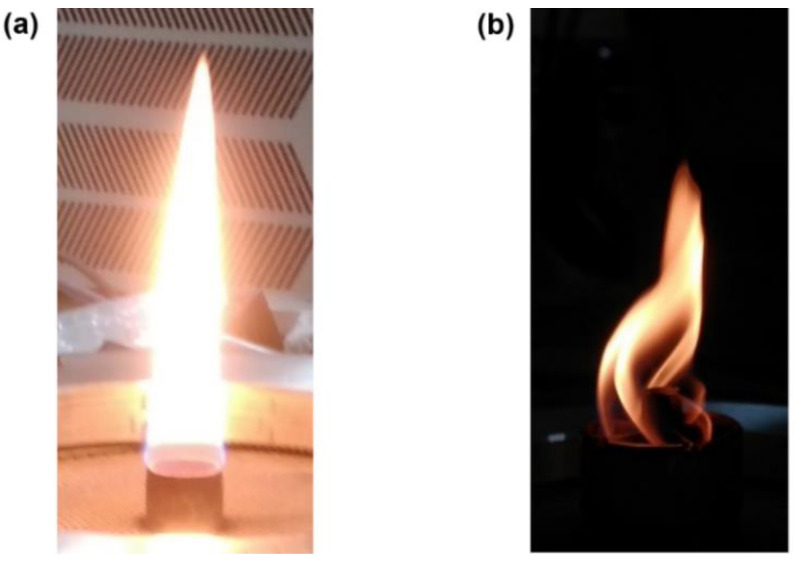
(**a**) Photograph of symmetric flame generated by a Gülder burner. (**b**) Photograph of asymmetric flame generated by mounting of a swirler on top of the burner nozzle shown in (**a**).

The Bunsen-type burner is chosen as it produces a laminar and axisymmetric non-premixed flame (see Figure 3a), which is well characterized in terms of soot formation [35,36,37,38]. It consists of a 10.9 mm internal diameter fuel injection tube and a co-annular 100 mm internal diameter airflow duct. Ethylene flow is metered using a mass flow controller (MC-5SLPM-D/5M, Alicat Scientific Inc., Tucson, AZ, USA) with a ±0.05 standard liter per minute (slpm) reading uncertainty. The airflow is measured using a rotameter (Aalborg, USA) with a ±8 slpm reading uncertainty. The fuel flow is fixed to 0.16 slpm and the co-flow of air is set to 200 slpm. An asymmetric flame (see Figure 3b) is obtained by inserting a swirler (diameter of 30 mm with five blades having 45° blade angle) on top of the burner nozzle. A wider flame compared to the symmetric flame was observed and, therefore, the fuel flow is slightly reduced here to 0.15 slpm. The co-flow of air is set to 160 slpm. It is done on purpose to restrict the whole area of the flame within the same field-of-view of the camera (as for symmetric flame). Less soot production is observed in the flame in terms of lower LII intensity due to the reduction in mixing time. This leads to trapping of the soot at the bottom of the swirler. Finally, to minimize the flickering of the flame, the complete experimental setup is covered with an enclosed housing.

## 4. Results

### 4.1. Simultaneous PAH-LIF and Soot-LII Imaging

**Figure 4:**
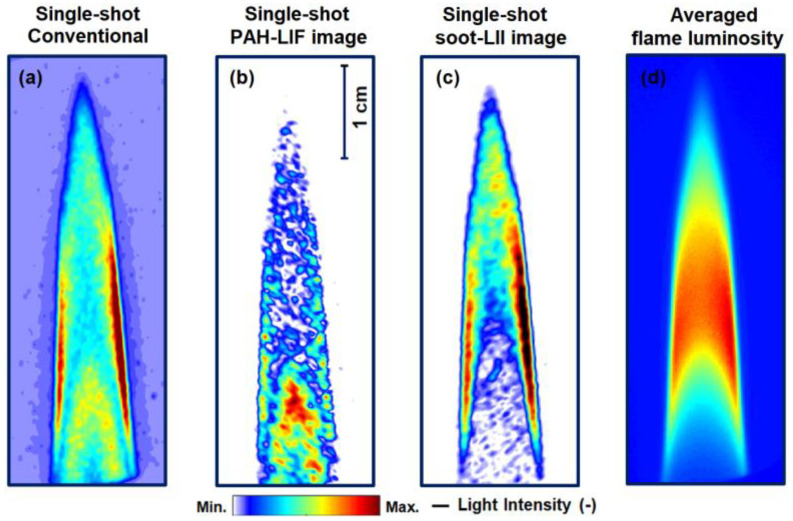
(**a**) “Conventional” (0th order components of FFT in Figure 1b) single-shot processed image of the flame, reconstructed after low-pass filtering of (LIF + LII) component in Figure 1b. (**b**) Processed single-shot qualitative PAH-LIF image after applying the lock-in algorithm on the FRAME image 1 in Figure 1a. Similarly, in (**c**), a processed single-shot qualitative soot-LII image is extracted. In (**b**,**c**), compared to (**a**), the image contrast is enhanced due to the suppression of stray lights and flame luminosity. Extinction of laser in the flame is evident from right to left in (**a**,**c**). However, in (**d**), some axis asymmetry between the right and left side of the flame is seen as well in terms of averaged flame luminosity, which is recorded without the lasers. This could be due to the weak flickering of the flame. Images are intensity-adjusted to pronounce the gradients of the probed species.

Figure 4 shows the single-shots of the central plane of the processed “conventional” image in (a), isolated PAH-LIF image in (b), and isolated soot-LII image in (c) extracted after the post-processing of the unprocessed FRAME image shown in Figure 1a. First, the symmetric flame is analyzed. The “conventional” image contains the data of merged LIF + LII signals. The flame luminosity and laser-induced reflections are also present as a background signal. In Figure 4b,c, isolated signals are presented, showing improved contrasts than in Figure 4a due to the suppression of unwanted stray lights. In Figure 4a,c, the extinction of light from right to left can be seen. Furthermore, some axis asymmetry in the flame is also observed in an averaged flame luminosity image (300 single-shots) in Figure 4d, which is a line-of-sight integrated signal without laser illumination. Note that the color maps of the images are individually intensity-adjusted and are non-normalized. Furthermore, the light intensity signals outside of the flame region are close to zero in the “non-conventional” images (Figure 4b,c) due to the suppression of the background i.e., flame luminosity and stray light in the lock-in algorithm. Thus, no intensity threshold is required. It is important to mention that the soot-LII signal has a non-linear dependence on the laser fluence [15,20]. Therefore, the modulated intensity-based excitation (532 nm wavelength) may generate modulated soot-LII signals lying between the plateau and the non-plateau regime of soot LII- laser fluence relation [15,20], which needs to be further investigated, especially for the quantitative analysis in terms of soot volume fraction. The present qualitative study assumes that the modulated laser fluence-LII signal lies within the plateau region. Throughout the experiments here, the averaged incident laser fluence for the laser-induced incandescence generation from the soot particles is ~ 0.27 J/cm^2^. This fluence value lies within the LII plateau region of ~0.25–0.28 J/cm^2^, for 532 nm excitation in an ethylene/air co-flow diffusion flame in an earlier work [42].

**Figure 5:**
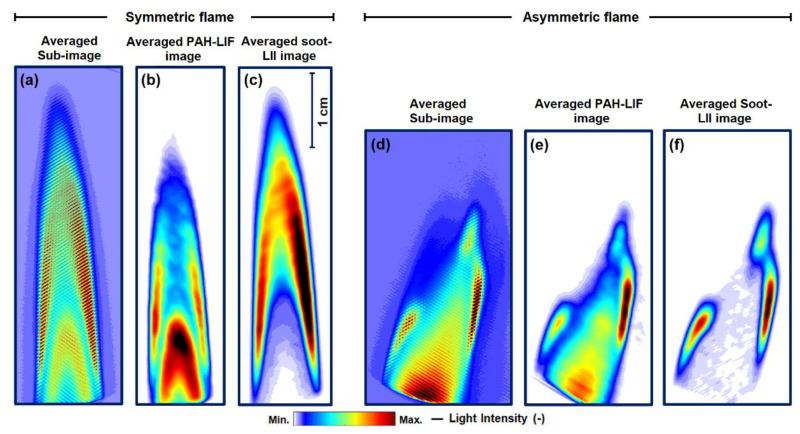
Symmetric flame: (**a**–**c**) Averaged images of the central plane corresponding to single-shots in Figure 4a–c. (**a**) The unprocessed sub-image, (**b**) processed PAH-LIF, and (**c**) processed soot-LII. Asymmetric flame: (**d**–**f**) shows the unprocessed averaged sub-image (not from the central plane) in (**d**), isolated PAH-LIF in (**e**) and isolated soot-LII in (**f**). While the qualitative PAH remains separated from LII in the innermost zone, it exists together with LII in the internal zone and secondary reaction zone. The laser extinction can be seen on both PAH (left to the right) and LII (right to the left).

Figure 5a shows an averaged modulated FRAME sub-image of the central plane of the symmetric flame. It is deduced from 300 single-shot images (similar to Figure 1a). Figure 5b,c show the corresponding isolated averaged PAH-LIF and averaged soot-LII images, respectively. Compared to single-shot images in Figure 4a–c, the averaged images exhibit less noise and “smoothed” intensity gradients. A minor loss in spatial resolution for the FRAME measurements for the averaged images is nearly unnoticeable. An averaged sub-image of the asymmetric flame is given in Figure 5d, and their corresponding isolated averaged PAH-LIF and soot-LII images are shown in Figure 5e,f, respectively. Note that the flame asymmetry is achieved here by mounting of a swirler on top of the burner nozzle (see Section 3.2, Figure 3b). The asymmetric flame images are not taken at the central plane as in Figure 5a. They are chosen from a different plane with high LII and LIF signals and large gradients to demonstrate the separation of two signals using the FRAME technique.

In the symmetric flame, the soot and PAH signals are again well separated within the flame. When comparing the single-shots of the PAH-LIF (Figure 4b) to the soot-LII image (Figure 4c), it is observed that the soot is distributed in the outer zones of the primary combustion zone and in the center of the secondary combustion zone. The majority of PAH occurs in the inner region of the flame and exists along with soot at the edges of the secondary reaction region. Similarly, in the averaged images in Figure 5b,c, PAH and soot are found together towards the outer edges. In the asymmetric flame, the PAH in Figure 5e is distributed throughout the flame, while soot is found in the outer region. For this flame, the production of soot is not as “efficient” as in the symmetric flame due to the faster mixing of fuel and air. This is also indicated by the lower PAH-LIF signal. On the right side, the LII signal is more “lifted” from the burner, which is due to the existing swirl in the flame. In general, the LII signal does not show any line pattern or non-monotonous behavior that would have been introduced by the different local fluence due to the light sheet modulation. Inversely, the LII signal is very homogeneous e.g., in the high-intensity region, which implies that quantitative measurements of the soot volume fraction should also be possible with appropriate calibration (e.g., by laser extinction measurements). Finally, to check any possible interferences between PAH-LIF and Soot-LII signals, individual images of only-LIF and only-LII signal are recorded—by keeping only one laser source in operation (see Appendix A). For both LIF-LII, the individual images are found similar to the reconstructed images in Figure 5b,c. For the axisymmetric flame, the qualitative distributions of both PAH-LIF and soot-LII are found to be matching in general to data in the literature [18,20,21]. For example, in [22,23,44,45] in ethylene-air non-premixed co-flow flames, PAHs are found to occur in an inner region, near the burner exit, and are surrounded by the soot region.

### 4.2. Simultaneous OH-LIF and Soot-LII Imaging

**Figure 6:**
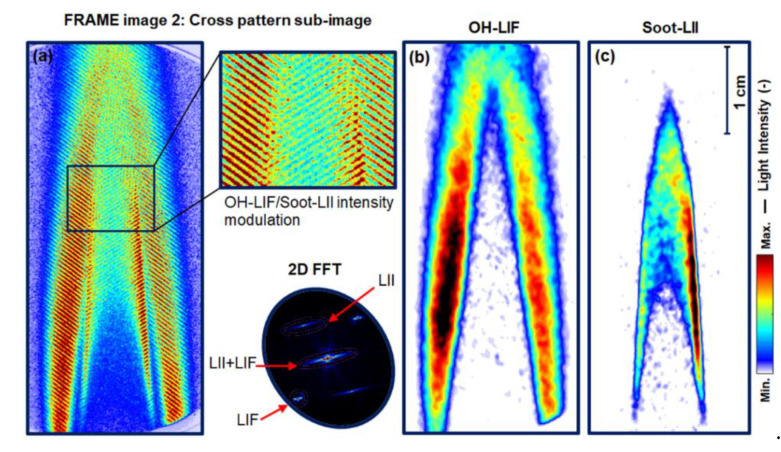
(**a**) Single-shot FRAME image: cross-patterned unprocessed sub-image and its FFT consisting of modulated signals of OH-LIF and soot-LII. Processed OH-LIF image in (**b**,**c**); the soot-LII image of the flame is extracted using the lock-in algorithm in (**a**). The signal reduction due to laser extinction is seen here from left to right in OH-LIF and from right to left in soot-LII. Qualitatively, most of the OH occurs in the reaction zone and soot-oxidation zone of the flame, whereas the soot is distributed in the internal zone and the secondary reaction zone.

Figure 6a shows the unprocessed single-shot FRAME image consisting of modulated OH-LIF and soot-LII signals along with a magnified view of this cross-patterned sub-image with its 2D-FFT. The OH radical exists especially in the outer flame region in which heat release and soot oxidation occur due to increased oxygen concentration. In the FFT image, it can be seen that individual fluorescence and incandescence modulations appear at different locations as the first-order peaks, while (LIF + LII) appears as the 0th order peak. Therefore, by using the lock-in algorithm (see Section 2, Figure 1), OH-LIF and soot-LII signals in Figure 6a can be isolated. The corresponding processed single-shots of isolated OH-LIF and soot-LII images are given in Figure 6b,c, respectively. Similar to the results in Section 4.1, the contrast of the processed images is enhanced due to the suppression of the stay light intensity. Furthermore, the extinction of signal intensity is seen in both OH-LIF (from left to right) and soot-LII (from right to left). Here, soot-LII in Figure 6c is lower in terms of intensity in comparison to Figure 4c due to the lower gain set for the intensifier (see Section 3.1).

**Figure 7:**
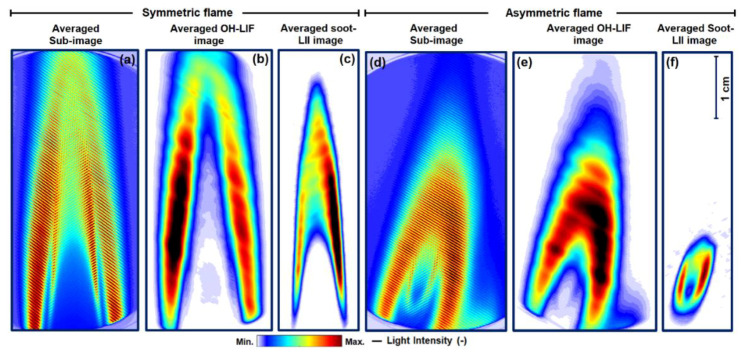
Symmetric flame: (**a**–**c**) averaged images corresponding to single-shots in Figure 6a–c. (**a**) Unprocessed sub-image, (**b**) processed OH-LIF, and (**c**) processed soot-LII. Asymmetric flame: the unprocessed averaged sub-image (different plane from Figure 5d and Figure 7a) is shown in (**d**), isolated OH-LIF in (**e**), and isolated soot-LII in (**f**). The swirled diffusion flame is characterized by a more compact flame shape leading to lower soot-LII signals compared to the Bunsen-type flame.

An averaged sub-image of the symmetric flame is displayed in Figure 7a. It is extracted from 300 single-shot images. The corresponding isolated averaged OH-LIF and averaged soot-LII images are shown in Figure 7b,c, respectively. Most of the OH is distributed on the outer edges, in the reaction region, and soot-oxidation region of the flame, whereas the soot is distributed in the central layer. An averaged unprocessed sub-image of the asymmetric flame (different plane from Figure 5d and Figure 7a) is given in Figure 7d, while their corresponding isolated averaged PAH-LIF and soot-LII images are shown in Figure 7e,f, respectively. Some line pattern is still visible in OH-asymmetric image mainly due to filtering. Throughout the image processing, the Gaussian filter size is kept the same (σ = 0.12) for a fair comparison. The lines disappeared from the OH images (in Figure 7e) when the filter size is reduced. Finally, it is worth mentioning that OH and soot-LII or PAH and soot-LII images can also be extracted from the unprocessed cross-patterned sub-images using an “unconventional approach” known as “first-order peak detection,” which is demonstrated in detail elsewhere [32]. In this study, the lock-in algorithm and the 1st order peak detection yielded identical 2D maps of probed species (not shown here). Finally, the minor loss in the spatial resolution is more visible in single-shot images in comparison to the averaged images.

## 5. Conclusions

The planar multispecies imaging technique FRAME was applied for the first time for simultaneous qualitative imaging of two species in a flame on a single camera without the use of an image doubler or additional cameras. It is based on structured illumination imaging, where the intensity of the laser sheet is spatially modulated. A Bunsen-type diffusion flame and an asymmetric swirl diffusion flame of ethylene/air were studied. First, simultaneous PAH and soot distribution have been mapped, and then 2D maps of OH and soot were imaged. The isolation of two signals from one image and enhanced contrast comes at a cost of very minor loss of spatial resolution in the resulting images. In comparison to the Bunsen-type flame, the swirled diffusion flame is characterized by a more compact flame shape due to increased air entrainment leading to lower PAH-LIF and soot-LII signals. However, a quantitative 3D analysis of the soot volume fraction is necessary for deeper insights into the soot formation and oxidation of these complex transient flames, which will be part of future studies. For this purpose, calibration procedures utilizing light extinction measurements will be employed to convert the qualitative data into images of quantitative soot volume fraction. Investigations will focus on the correlation of modulated laser fluence and soot-LII signal. Furthermore, a comparison between different configurations of FRAME optical setup for the reconstruction of the resulting LIF-LII images from a single image and the quantitative comparison of stray-light reduction in FRAME and “conventional” detections are also under investigation.

## Figures and Tables

**Figure A1:**
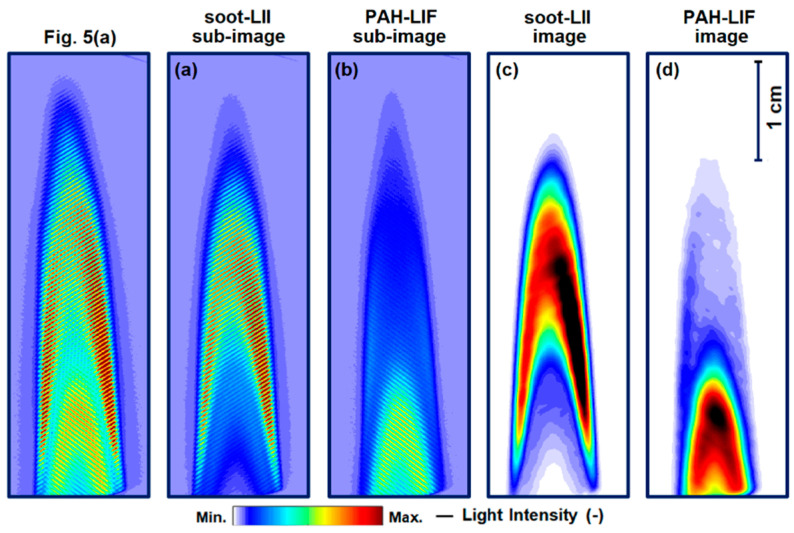
Averaged sub-image of symmetric flame containing modulated signals of both PAH-LII given in Figure 5a. In (**a**,**b**), the unprocessed averaged sub-images (300 single-shots, central plane) of only soot-LII signal and only PAH-LIF are given, respectively. Therefore, only one species is excited at a time and not simultaneously. In (**c**,**d**), the processed soot-LII and PAH-LIF corresponding to (**a**,**b**), respectively, are given.

## Appendix A

Here, the isolation of soot-LII and PAH-LIF from one sub-image consisting of both signals (Figure 5a) is demonstrated in the central plane of the symmetric flame. In Figure A1a,b, averaged unprocessed sub-images (300 single-shots) of individual modulated LII and modulated LIF, respectively, are given. As described above, all the sub-images shown here are recorded one after another and not simultaneously. Therefore, the differences in the heights of the three sub-images are visible. For recording the sub-image in Figure A1a, the PAH-LIF laser source is blocked, and similarly, for Figure A1b, the soot-LII laser beam is blocked. The corresponding processed images of (a) and (b) are given in Figure A1c,d, respectively. In (c), a very similar distribution (see Figure 5c) of soot-LII is observed, however, with a reduced flame height. In (d), most of the PAHs are existing at the inner region near to burner exit, which is similar to the PAH-LIF distribution in Figure 5b. However, the flame height is lower and the PAH signal is lower on the outer edges, especially on the right side. This is because the modulation depth of the structured laser sheet has significantly reduced on the right side of the symmetric flame in (b).
